# Wired to Survive: How AML Cytogenetics Shape Apoptotic Dependence and Venetoclax Resistance

**DOI:** 10.3390/genes17091122

**Published:** 2026-09-15

**Authors:** Arnold Rojas, Sahil Jethi, Tulin Budak-Alpdogan, Manoj K. Pandey

**Affiliations:** 1 Department of Biomedical Sciences, Cooper Medical School of Rowan University, Camden, NJ 08103, USA; rojasa25@rowan.edu (A.R.); jethis97@rowan.edu (S.J.); 2 Department of Hematology, Cooper University Hospital, Camden, NJ 08103, USA; budak-alpdogan-tulin@cooperhealth.edu

**Keywords:** acute myeloid leukemia, cytogenetics, venetoclax, BCL-2 family proteins, BH3 mimetics, mitochondrial apoptosis, therapeutic resistance, targeted therapy

## Abstract

Acute myeloid leukemia (AML) is cytogenetically and phenotypically heterogeneous, and this diversity contributes to differences in how patients respond to therapies that target apoptosis. Venetoclax, a selective BCL-2 inhibitor, has been demonstrated to improve outcomes when combined with hypomethylating drugs (HMAs) such as azacitidine or decitabine; nonetheless, clinical trials have indicated that resistance and recurrence are prevalent. This review examines the current evidence linking chromosomal abnormalities and cellular differentiation state to mitochondrial apoptotic pathways, with an emphasis on how these factors influence dependence on certain anti-apoptotic BCL-2 family proteins. We summarize how specific cytogenetic subtypes and high-risk groups (including monosomy 7/del(7q) and complex karyotype/TP53-altered AML) frequently show stress-adaptive signaling and reliance on multiple anti-apoptotic pathways, which can limit the durability of response to BCL-2 inhibition. Lineage-associated dependencies are also examined, such as monocytic differentiation (which leads to increased MCL-1 reliance) and erythroid/megakaryocytic differentiation, which has been associated with increased BCL-XL dependence and venetoclax resistance. Finally, we discuss the therapeutic implications of dependence mapping, including venetoclax combinations and direct MCL-1/BCL-XL targeting, and propose promising biomarker strategies that can detect dependence shifts early and guide appropriate treatment selection.

## 1. Introduction

Acute myeloid leukemia (AML) is an aggressive hematologic cancer that is typically fatal within weeks or months if left untreated. It has a cytogenetically heterogeneous profile, in which several chromosomal aberrations can shape its clinical behavior and therapeutic response. Diagnostic frameworks developed by European Leukemia Net (ELN) place cytogenetics at the center of AML risk classification and treatment planning since specific chromosomal changes are linked to distinct AML subtypes and clinical outcomes [1]. At the same time, there has been a shift towards lower-intensity therapies for older, medically unfit patients since previous risk classifications were developed based on data from younger, more medically fit patients receiving higher-intensity treatments. This has led to the cytogenetic risk categories being reinterpreted using newer treatment regimens, not just intensive chemotherapy [2]. This creates an opportunity to study the different cytogenetic groups and how they interact with today’s newer therapies.

These newer, lower-intensity medicines have altered the treatment paradigm for patients who were previously ineligible for intensive chemotherapy regimens. Venetoclax, a selective BCL-2 inhibitor, received FDA approval in 2020 when used in combination with hypomethylating agents such as azacitidine or decitabine [3]. During phase 3 of the VIALE-A trial, patients ineligible for intensive chemotherapy had higher overall survival and rates of remission with azacitidine and venetoclax compared to azacitidine alone [4]. Biological studies have suggested that part of the reason why azacitidine–venetoclax is effective is due to their disruption of leukemic stem cell metabolism, thus linking to apoptotic pathways, which vary across the AML risk categories [5]. These developments are clinically important, but patient response can vary and eventually lead to a relapse, indicating that the main challenge here is understanding why some patients respond well while others relapse with resistance after initially responding.

Mechanistically, venetoclax targets the anti-apoptotic (pro-survival) role of BCL-2 mainly at the mitochondria. When AML cells emerge that rely on other anti-apoptotic proteins such as MCL-1 or BCL-XL, the BCL-2 inhibition can be ineffective and lead to the survival of cell populations with different maturity or survival pathways. Along these lines, one study documented that monocytic-differentiated AML is more resistant to azacitidine–venetoclax due to transcriptional changes that reduce BCL-2 and upregulate MCL-1 survival pathways, leading to selective growth of a monocytic differentiation at relapse [6]. Similarly, erythroid or megakaryocytic leukemic cell lines have also shown resistance to venetoclax by switching survival dependence to BCL-XL [7]. Because survival dependency is linked to a cell’s differentiation state, resistance to venetoclax can be seen as a predictable, lineage-associated phenomenon. Conversely, relapse can also be seen outside these lineage patterns, as seen in populations with acquired mutations to BAX and resistance to BH3 proteins during venetoclax therapy [8]. These relapses highlight that resistance can potentially arise from any part of the survival pathway.

These findings suggest a link between cytogenetics and apoptotic dependence, since chromosomal aberrations can change the transcriptional and signaling pathways that directly affect cell differentiation and reliance on the BCL-2 protein family. The question is whether cytogenetic features can predict which survival pathway a patient’s leukemic cells rely on and whether the cells will be sensitive to venetoclax or prone to resistance through MCL-1 or BCL-XL resistance. Recent studies have suggested that this approach is feasible, showing that BCL-2 protein expression in leukemic stem cells can predict clinical response to azacitidine–venetoclax therapy [9]. Therefore, the literature supports the idea that cytogenetic features shape cell phenotypes and signaling, which in turn determine apoptotic dependence and explain why venetoclax has variable clinical responses.

This review will therefore focus on how cytogenetic aberrations and their differentiation states impact BCL-2 protein family dependence in AML, emphasizing how venetoclax effectiveness is reduced and escape pathways develop around MCL-1 or BCL-XL. The current AML classifications and their main cytogenetic categories will first be summarized and linked to the mitochondrial apoptosis pathway targeted by BH3 mimetics [1,2]. Then, the cytogenetic and phenotypic AML subsets will be examined to explain why some respond to venetoclax and others do not, with a focus on BCL-XL-dependent and monocytic cells that are less dependent on BCL-2 [6,7]. Finally, the review will summarize the key resistance pathways, including shifts in differentiation state and impaired apoptosis, and will explore potential treatment strategies such as combination regimens to limit MCL-1 or BCL-XL escape and biomarkers to monitor therapeutic efficacy [8,9].

## 2. AML Cytogenetics

Cytogenetic characterization is critical to AML classification because each abnormality can be classified into a disease subtype that can guide treatment selection, prognosis, and even the risk of relapse. Current ELN guidelines match chromosomal aberrations with certain gene mutations to stratify AML subtypes into favorable, intermediate, and adverse-risk groups, based on outcome data collected from large cohorts [1,10]. One study created an ELN genetic risk classification that was proposed to reflect patients receiving less-intensive therapies such as venetoclax, recognizing that a shift to less-intensive treatment has changed the prognostics of subtypes [2]. More recently, the WHO 2022 and ICC 2022 have released guidelines that reinforce the idea that cytogenetic lesions found in leukemic cells are not just prognostic but disease-defining features [11,12].

In these classification systems, chromosomal aberrations are matched into distinct AML subtypes that have unique biological characteristics. For example, the abnormalities t(8;21)(q22;22.1) and inv(16)(p13.1q22) are categorized as favorable risk. Other abnormalities, such as MLLT3: KMT2A, are generally considered intermediate risk with their own subgroup defined by a unique transcriptional profile and clinical outcome. At the adverse end of classification, the ELN and other organizations classify complex karyotypes, unbalanced abnormalities such as -5/5q- or -7/7q-, and high-risk rearrangements such as inv(3)(q21.3q26.2) as cytogenetic features with poor clinical behavior [1,10]. In their 2022 framework, the WHO formally recognized a wider range of genetically defined AML subtypes and abolished the mandatory 20% blast requirement. This underscores that cytogenetic aberrations can define AML biology even when morphology was previously borderline [12,13].

In terms of the pathogenesis, chromosomal aberrations drive leukemic growth through three main mechanisms: (i) the formation of fused genes that disrupt normal transcription and block differentiation, (ii) changes in genetic material that directly impact tumor suppressor and apoptotic pathways, and (iii) genetic instability, as pointed out by the complex karyotypes and those with TP53 dysfunction and thus resistance to therapy [1,14] These kinds of cytogenetic lesions impact how leukemic cells behave by locking them into a specific differentiation, promoting uncontrolled cell growth, and dysregulating their regulatory mechanisms. Clinically, this matters when it comes to choosing therapies because characterizing the cytogenetic features reflects a broader cell pathway dysfunction rather than a single gene change [1,2].

The cytogenetic changes seen in different AML subtypes provide clues about which survival pathway leukemic cells depend on and their implications for treatment choice. These chromosomal abnormalities can impact transcriptional and signaling machinery cells rely on to regulate apoptotic pathways, determining whether leukemic cells depend on BCL-2, MCL-1, or BCL-XL for survival [1,12]. These connections help explain why venetoclax works well in some AML subtypes but loses effectiveness or becomes resistant in others, since the cytogenetics shape which anti-apoptotic pathway leukemic cells depend on to develop resistance [1,2].

## 3. Apoptosis and BH3 Mimetics in AML

The BCL-2 protein family regulates what is called the intrinsic mitochondrial pathway of apoptosis, which permeabilizes the mitochondrial membrane and leads to cell death. The anti-apoptotic BCL-2 protein family, including MCL-1 and BCL-XL, keeps the mitochondria intact by neutralizing pro-apoptotic proteins such as BAX and BAK. When these pro-apoptotic proteins are no longer inhibited, this leads to membrane permeabilization. Regulators of these pro-apoptotic proteins include BH3-only proteins, which can sense cellular stress to either directly activate BAX/BAK and promote apoptosis or neutralize them [15,16]. These regulatory pathways are frequently disrupted in AML, both because of altered BCL-2 expression and changes to cell differentiation and signaling that make cells more resistant to apoptosis [17].

One way to characterize this resistance to apoptosis is mitochondrial priming, which refers to a cell’s “readiness” for triggering apoptosis via membrane outer membrane permeabilization (MOMP). BH3 profiling is one functional assay that can be used to measure this apoptotic threshold by exposing the mitochondrial membrane to BH3 peptides and measuring the MOMP. Studies have shown that the use of BH3 profiling can identify apoptotic deficits and cells reliant on BCL-2 for survival, including cell susceptibility to BCL-2 antagonists [18]. This technique is important in AML since what matters is not just which protein is more apoptotic, but rather which protein is neutralizing pro-apoptotic signals.

Building on this concept, AML cells often rely on a dominant anti-apoptotic protein to inhibit pro-apoptotic signals and maintain survival. BH3 mimetics take advantage of this vulnerability by mimicking natural BH3-only proteins and binding to anti-apoptotic proteins, freeing them to activate a cell’s apoptotic pathway via BAX and BAK and cause cell death [16,19]. However, the BCL-2 protein family can perform similar functions, allowing AML cells to change their reliance on different anti-apoptotic proteins, moving from BCL-2 to either MCL-1 or BCL-XL. This concept, known as buffering or dependency switching, can explain why venetoclax often demonstrates a strong initial clinical response that then wanes. It also highlights the importance of measuring levels of multiple anti-apoptotic proteins instead of just BCL-2. One notable study used flow cytometry to measure expression of multiple anti-apoptotic proteins in leukemic stem cells to create a combinational score that predicted azacitidine–venetoclax response, emphasizing that measuring multi-protein expression has prognostic implications [9].

Venetoclax is the most clinically mature and widely approved BH3 mimetic in AML that selectively targets BCL-2. Early preclinical studies demonstrated this when venetoclax targeting of BCL-2 led to cell death, with BH3 profiling predicting its efficacy [20]. Later studies showed that venetoclax monotherapy had modest activity in AML patients with relapsed or unfit AML, consistent with the fact that many AML cases are not solely dependent on BCL-2 [21]. This has led to the current consensus that venetoclax efficacy can be potentiated when in combination with hypomethylating agents. Research illustrates that combination therapy is biologically sound since azacitidine leads to disruption in leukemic stem cell metabolism and oxidative phosphorylation, in addition to improved clinical efficacy [5]. Other studies have suggested that these hypomethylating agents improve efficacy by “priming” AML cells for venetoclax treatment by upregulating pro-apoptosis factors such as NOXA, weakening the leukemic cell’s ability to develop escape pathways and promoting apoptosis [22,23].

A constant result across research is that the major anti-apoptotic protein on which AML cells rely is not stable but can vary over time. This means that to fully understand cell survival requires characterization before and during treatment. The use of dynamic BH3 profiling has been shown to better detect apoptotic dependence before and during treatment when compared to standard BH3 profiling that captures a snapshot of the apoptotic state. Another approach measures protein expression ratios, such as BCL-2 versus MCL-1 or BCL-XL, in leukemic stem cells to determine survival dependence and is easier to apply to clinical studies [9]. These assays establish the basis for linking the cytogenetic and phenotypic AML features to venetoclax response and their escape mechanisms.

## 4. Cytogenetic and Phenotypic Drivers of BCL-2 Dependence

### 4.1. Core-Binding Factor (CBF)-AML

**Figure 1:**
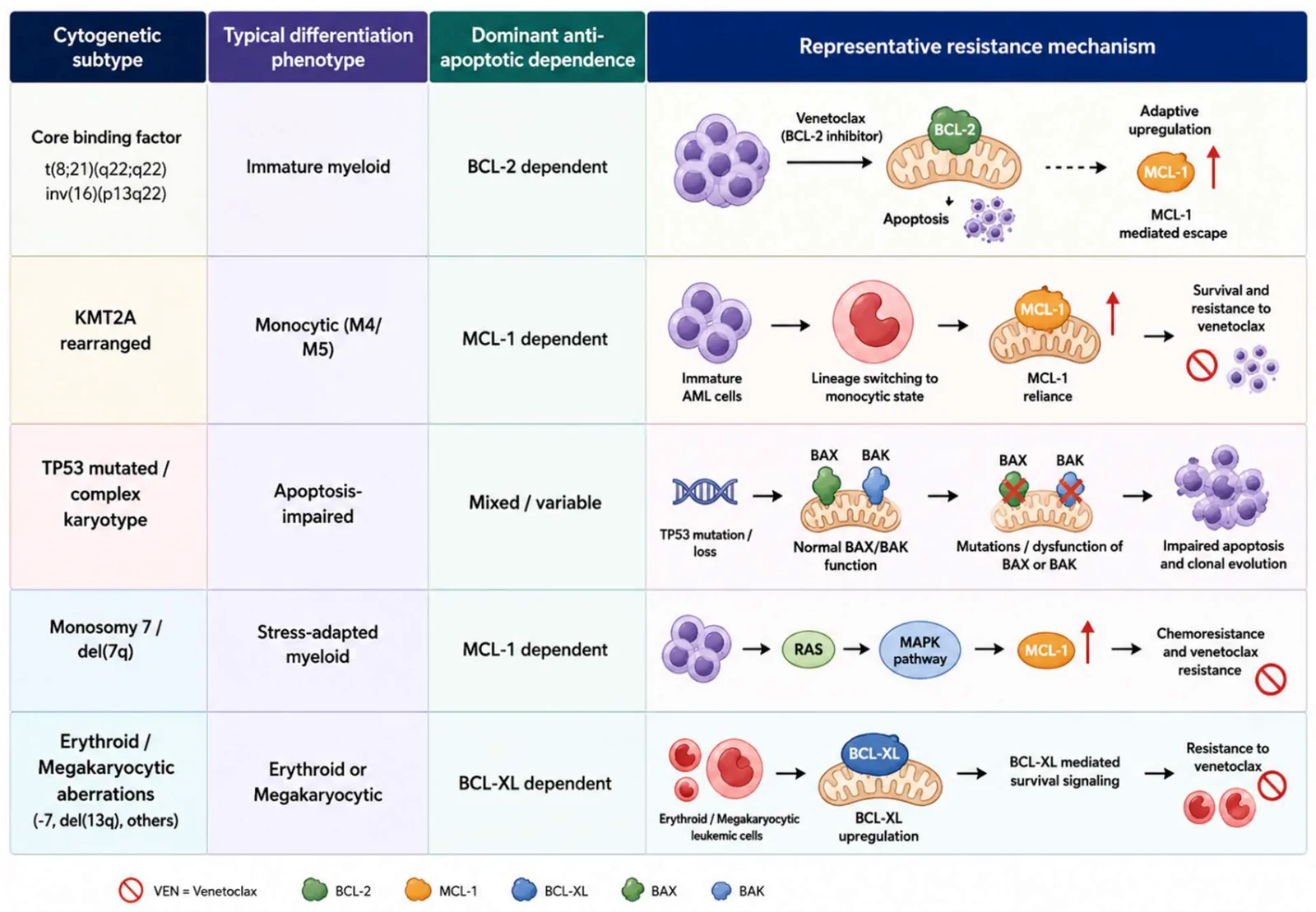
Cytogenetic and differentiation-dependent apoptotic dependencies in AML. Cytogenetic abnormalities and differentiation states shape anti-apoptotic dependencies and influence venetoclax response in AML. BCL-2–dependent subtypes are generally more susceptible to venetoclax, whereas lineage switching, MCL-1 or BCL-XL dependence, impaired BAX/BAK function, and RAS–MAPK signaling can promote resistance [14,24,25].

CBF-AML is a subtype defined by the chromosomal rearrangements t(8;21) and inv(16) (Figure 1). These cytogenetic lesions are classified by the ELN as a favorable-risk group, although co-mutations and other genetic factors can influence prognosis too. Clinical data indicate that CBF-AML is very responsive to chemotherapy, but it is important to differentiate between t(8;21) and inv(16) because clinical outcomes differ between these lesions. Indeed, studies have shown differences among these aberrations when it comes to relapse risk and long-term outcomes [10].

Most evidence for venetoclax with hypomethylating agents in CBF-AML comes from studies that also offer insight into BCL-2 dependence in this subgroup. In the large CBF-AML cohort study by Zhang et al., responses to venetoclax plus hypomethylating agents were better in the inv(16) group, but clinically suboptimal in the t(8;21) group. Additional studies also support that while venetoclax combination therapy has shown clinical efficacy in select CBF-AML patients treated with low-intensity regimens, BCL-2 dependence cannot be generalized across the CBF subtype [26,27]. Thus, it is reasonable to conclude that CBF-AML is vulnerable to BCL-2 inhibition, but this vulnerability can be variable and influenced by other biological factors rather than just BCL-2 dependence [26,27].

### 4.2. KMT2A-Rearranged AML

This subtype of AML is defined by oncogenic KMT2A gene fusions that disrupt normal gene regulation, leading to inappropriate activation of MEIS1 and HOXA cluster genes (Figure 1). Clinical data have shown aggressive clinical behavior and increased risk of relapses in this subtype [28]. The KMT2A fusions often recruit helper proteins, such as menin, that keep leukemic cells in a stable epigenetic and transcriptional state to support leukemic cell survival and growth. This underscores that KMT2A-rearranged AML is driven by an altered epigenetic and transcriptional state rather than a single downstream signaling pathway [28,29].

This altered transcriptional state also affects mitochondrial survival pathways. In primary patient samples, KMT2A-rearranged AML has shown partial sensitivity to MCL-1 inhibition, consistent with a reduced reliance on BCL-2-mediated survival pathways [30]. This helps explain why venetoclax therapy alone is not sufficient, since pro-apoptotic proteins are sequestered by MCL-1 or BCL-XL instead of BCL-2, thus allowing cells to evade BCL-2 inhibition. Conversely, one preclinical trial has demonstrated that co-targeting of BCL-2 and MCL-1 can restore apoptotic wiring and overcome venetoclax resistance, indicating that MCL-1 represents a rational co-target when BCL-2 inhibition alone is insufficient [31].

KMT2A-rearranged AML commonly exhibits monocytic differentiation, a lineage state associated with reduced sensitivity to venetoclax-based therapies. Clinical studies with azacitidine–venetoclax have demonstrated that treatment efficacy is correlated to a cell’s differentiation state, with monocytic lineages at highest risk for resistance and relapses [6]. This cell lineage distinction is relevant because this subtype may have reduced dependence on BCL-2 at baseline, and starting venetoclax-based therapy may cause these leukemic cells to adapt even further by shifting survival dependence to other anti-apoptotic proteins such as MCL-1 or BCL-XL [6].

Two therapeutic strategies are being investigated that can be explored for this subtype: (i) disrupting KMT2A-driven transcriptional alterations or (ii) inhibiting secondary anti-apoptotic pathways. An example of the first strategy has been explored in one preclinical study, where menin–KMT2A inhibition synergizes with azacitidine–venetoclax therapy in KMT2A-rearranged AML models, supporting the idea of potential triple therapy [32,33]. This has been translated into a clinical study, where the menin inhibitor revumenib has been combined with azacitidine–venetoclax in adults with KMT2A-rearranged AML, demonstrating early feasibility and providing a clinical link between HOXA/MEIS1 transcriptional alterations and a rational triplet therapy approach [34].

### 4.3. Monosomy 7/Deletion 7q and Other High-Risk Groups

The monosomy 7/del(7q) AML subtype is classified as an adverse-risk group that arises from prior disease or treatment and survives by activating a broad range of stress and survival pathways, rather than just one oncogenic driver (Figure 1) [35]. In a study by Shah et al., chromosome 7 abnormalities in venetoclax-treated cohorts were associated with shorter survival and overall inferior outcomes, suggesting that BCL-2 dependence is weakened in high-risk cytogenetic groups [36]. These findings have been emphasized in other literature regarding venetoclax-resistant AML, where adverse-risk groups use secondary anti-apoptotic channels including RAS/MAPK and MCL-1 to survive [37].

RAS/MAPK signaling has been shown to promote MCL-1 stability via ERK-mediated phosphorylation, allowing leukemic cells to bypass BCL-2 inhibition under stress [38]. A study by Zhang et al. showed that the RAS/MAPK pathway promoted resistance to venetoclax through upregulation of MCL-1, linking this pathway to an escape pathway. All these findings are clinically relevant because they support the idea of combination therapy in high-risk cytogenetic groups such as monosomy 7/del(7q). Combining venetoclax with MAPK inhibition to prevent MCL-1 stabilization, or targeting MCL-1 directly, may help overcome BCL-2 resistance [37].

### 4.4. Complex Karyotype and TP53-Altered AML

Complex karyotype AML is a cytogenetically unstable subtype that is frequently associated with TP53 alterations and is classified as adverse risk according to ELN criteria [1]. Much like the monosomy 7/del(7q) AML subtype, this category demonstrates a broad range of apoptosis-resistant pathways rather than survival on one anti-apoptotic protein. The disruption of TP53 impairs normal stress responses, leading to reduced mitochondrial priming and increased stress tolerance (Figure 1). As a result, activation of the mitochondrial apoptosis pathway is impaired due to disruption of BAX/BAK, reducing the efficacy of therapies that rely on the intrinsic apoptotic pathway for cell death [8,39].

With venetoclax-based therapy, these cytogenetic lesions are associated with responses that may occur but are typically not sustained compared to TP53-wild type AML. Among the VIALE-A patients with cytogenetically confirmed poor-risk subtype, azacitidine–venetoclax therapy saw improved remission rates in TP53-altered AML but did not substantially improve the remission duration or overall survival compared to azacitidine alone, indicating a limited durability of response. Relapse following venetoclax therapy may occur through acquired genetic alterations that impair apoptotic signaling. In particular, inactivating BAX mutations can disrupt mitochondrial apoptosis, providing leukemic cells with a survival advantage under venetoclax-mediated selective pressure [8].

Collectively, these findings suggest two important implications for complex karyotype/TP53-altered AML. First, although venetoclax may provide initial therapeutic benefit, responses are often short-lived as leukemic cells acquire or select for resistance mechanisms, including increased dependence on alternative anti-apoptotic proteins such as MCL-1 or BCL-XL and impairment of BAX-mediated mitochondrial apoptosis. These adaptations can diminish BCL-2 dependence and reduce sensitivity to venetoclax [8,39]. Second, these observations highlight the need for therapeutic strategies that extend beyond BCL-2 inhibition by targeting parallel survival pathways and alternative cellular vulnerabilities to limit therapeutic resistance and disease escape [39].

### 4.5. Trisomy 8 and Normal Karyotype

Trisomy 8 and normal-karyotype AML illustrate a limitation of cytogenetics, as both are cytogenetically broad, heterogeneous categories in which apoptotic dependence, particularly BCL-2 reliance, is driven more by mutation status and cell state than karyotype (Figure 1) [1,10]. In real-world trials, cohorts with trisomy 8 were shown to have poorer survival with azacitidine–venetoclax therapy, suggesting that adverse co-features play a bigger role than trisomy 8 itself [40]. To better delineate the features that influence apoptotic dependency, functional and qualitative assays add value to BH3 profiling, including dynamic BH3 profiling, which measures mitochondrial priming and identifies which anti-apoptotic proteins a cell depends on, enabling the prediction of response to BH3 mimetics [41,42]. One study has shown that measuring multiple BCL-2 family proteins at the same time could predict which patients could respond to azacitidine–venetoclax therapy, providing a practical bridge from broad cytogenetic categories to treatments that target apoptosis dependence and improve venetoclax sensitivity [9].

### 4.6. Molecular Mutations and Venetoclax Resistance

RUNX1 mutations in AML exhibit diverse and context-dependent relationships with response to venetoclax-based therapy, and their significance in venetoclax resistance is unknown. In a frontline investigation of AML patients over the age of 60, RUNX1 mutations were found in 40% of those with refractory illness, indicating a possible link to treatment resistance. However, RUNX1 mutations were also found in patients who sustained long-term remissions, including those with co-occurring IDH2 or SRSF2 mutations. These findings suggest that RUNX1 mutation alone is unlikely to cause venetoclax resistance, and that its impact may be dependent on the larger molecular background and treatment situation [43,44].

Conversely, clinically meaningful responses to venetoclax-based combinations have been reported in relapsed and refractory RUNX1-mutated AML. From a mechanistic point of view, the effects of RUNX1 mutations can involve changes in ribosome biogenesis, cellular metabolism and differentiation, thus possibly affecting mitochondrial apoptosis dependence and susceptibility to BCL-2 inhibitors. Therefore, the correlation between RUNX1 mutations and venetoclax therapy effects depends on the clinical scenario, additional mutation burden, and apoptosis/metabolic characteristics of the leukemic cells themselves [45,46].

ASXL1 mutations in AML have also exhibited context-dependent relationships with response to venetoclax-based therapy, although they do not appear to be an established mechanism of venetoclax resistance. Preclinical studies indicate that ASXL1 mutations increase chromatin accessibility at the BCL2 locus, leading to increased BCL-2 expression and increasing reliance on BCL-2-mediated survival. Consistent with this approach, ASXL1-mutant cells showed higher apoptosis after venetoclax treatment as compared to genetically restored controls. Clinical trials have shown that patients with ASXL1-mutated AML and other myeloid malignancies respond well to venetoclax-based therapy; however, the strength of this connection varies by patient type and treatment scenario. Current research suggests that ASXL1 mutation may identify a physiologically different subgroup with enhanced BCL-2 reliance rather than conferring venetoclax resistance; nonetheless, its predictive significance as a clinical biomarker has to be validated [43,44,46].

### 4.7. Phenotypic Correlates with Cytogenetic Groups

Among AML subtypes, cytogenetic lesions are often associated with specific lineages. In contrast, resistance to BH3 mimetics often follows the differentiation state rather than the cytogenetics of the cell. While the cytogenetics predict which differentiation state the cells will adopt, venetoclax sensitivity largely depends on the apoptotic dependencies and other anti-apoptotic pathways maintaining cell survival [9,47].

Monocytic-differentiated AML is one example of a differentiation state being correlated to venetoclax sensitivity. In a study by Pei et al., paired patient and experimental samples treated with azacitidine–venetoclax therapy revealed selection of monocytic-differentiated cells at relapse, with resistance being linked to the intrinsic features of monocytic differentiation [6]. Previous studies have characterized monocytic AML as less dependent on BCL-2 and more reliant on alternative anti-apoptotic pathways, such as MCL-1, therefore reducing the effectiveness of BCL-2 inhibition [6,47]. These findings are consistent with other mechanistic studies showing that venetoclax resistance arises from reduced mitochondrial apoptotic priming and enhanced alternative anti-apoptotic pathways, rather than the emergence of new recurrent gene mutations [47,48].

Erythroid/megakaryocytic-differentiated AML also represents a distinct lineage state that follows a different anti-apoptotic dependence. A study by Kuusanmäki et al. showed that AML cells with erythrocytic and megakaryocytic differentiation had increased dependence on BCL-XL instead of BCL-2 when profiled [7]. Thus, increased resistance to venetoclax therapy is expected, since survival depends less on BCL-2 and more on BCL-XL, supporting evaluation of approaches that target BCL-XL in erythroid/megakaryocytic-differentiated cells [7].

Phenotype-associated dependencies also explain why targeting a single marker is inconsistent across these cohorts. Multiple studies have supported transitioning away from phenotype-based treatment and instead to therapies that target multi-protein dependence states, such as azacitidine–venetoclax therapy, which better capture broad apoptotic vulnerabilities across leukemic cell groups [9,47].

Overall, the studies have shown support for the idea that resistance is tied to differentiation state. Monocytic-differentiated AML shifts anti-apoptotic dependence away to MCL-1, and erythroid/megakaryocytic differentiation shifts dependence towards BCL-XL. Both phenotypes lead to reduced efficacy of targeted BCL-2 inhibition [6,7]. By integrating phenotypic findings with cytogenetics, this approach links cytogenetic categories to venetoclax outcomes and supports the idea for dependence-guided therapies discussed in subsequent sections [9,47].

## 5. Mechanisms of Resistance for BCL-2 Inhibition

### 5.1. Primary Resistance

**Figure 2:**
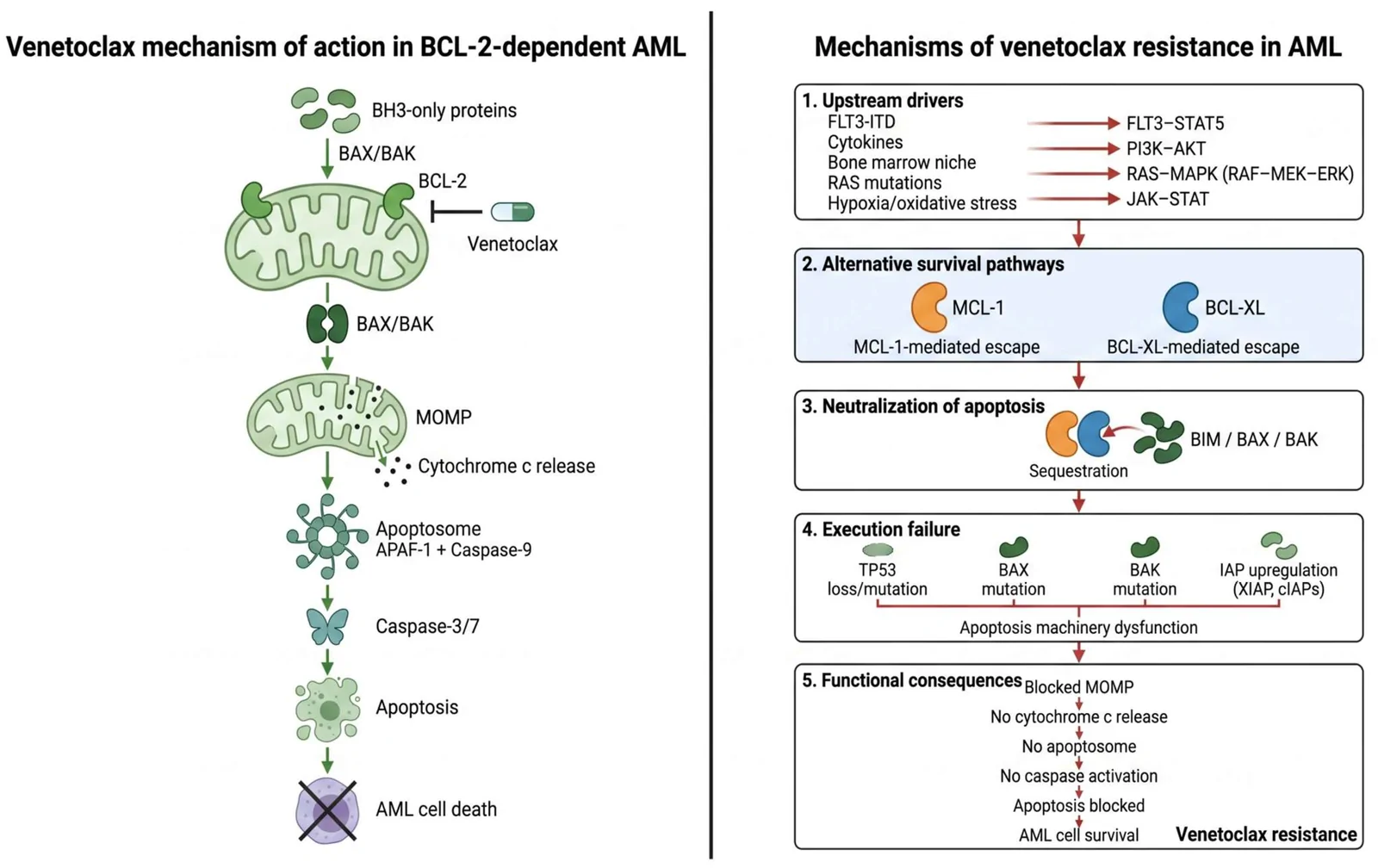
Mechanisms of venetoclax action and resistance in AML. Venetoclax inhibits BCL-2 and promotes BAX/BAK-mediated mitochondrial outer membrane permeabilization (MOMP), cytochrome c release, caspase activation, and apoptosis. Venetoclax resistance may arise through activation of upstream survival signaling, increased MCL-1 or BCL-XL dependence, sequestration of pro-apoptotic proteins, or defects in the apoptotic machinery [37,47,49,50,51].

Primary venetoclax resistance reflects a state in which BCL-2 inhibition is insufficient to induce mitochondrial apoptosis and ultimate cell death. Clinically, this is reflected in studies performed by Konopleva et al. that show modest activity with venetoclax monotherapy in refractory/relapsed AML. There was increased activity in leukemic cells with greater BCL-2 dependence, yet most patients showed minimal responses. This indicates that BCL-2 was not the main anti-apoptotic protein in these cells. This can be explained mechanistically, as resistance is often linked to cells using a broader array of anti-apoptotic pathways such as MCL-1 or BCL-XL, combined with lower mitochondrial priming (Figure 2). As a result, inhibiting BCL-2 does not create enough cell stress to trigger apoptosis. This is supported by the work done in one study, which integrated multiple biological and clinical data types and showed that assessing multiple BCL-2 dependency states, rather than a single one, more accurately captures venetoclax responsiveness [9].

Differentiation state is another key determinant of therapy effectiveness, as the key mechanism of resistance lies in a cell’s preexisting pathways rather than new recurrent gene mutations. As discussed earlier, monocytic-differentiated AML is less sensitive to azacitidine–venetoclax therapy, and biochemical analyses showed reduced BCL-2 expression and greater dependence on MCL-1, creating a baseline state where BCL-2 inhibition is not effective [9]. Meanwhile, erythroid- and megakaryocytic-differentiated AML cells offer a parallel case, instead relying on BCL-XL and being resistant to venetoclax, further illustrating that primary resistance arises from a shift in anti-apoptotic dependence away from BCL-2 [7]. Nonetheless, clinical prediction based on phenotype alone is not sufficient. Studies integrating cell lineage with multi-protein dependency measures show that primary resistance is best understood as a measurable baseline dependency state that can guide rational treatment combinations [9].

### 5.2. Adaptive Resistance Under Treatment

Leukemic cells may initially be responsive to venetoclax therapy but later adapt to venetoclax therapy to develop resistance and survive. This phenomenon occurs due to loss of BCL-2 dependence, driven by increased expression and reliance on other apoptotic proteins, such as MCL-1 and BCL-XL, which capture BH3-only activators and prevent BAX/BAK activation. In AML cell lines made venetoclax-resistant by prolonged drug exposure, resistant cells were observed to have upregulated MCL-1 and sometimes BCL-XL (Figure 2), resulting in a state in which BCL-2 was no longer effective to induce apoptosis [31,52]. Other mechanistic studies support this idea, showing that venetoclax resistance is often associated with signaling-related changes that upregulate dependence on MCL-1 and maintain mitochondrial function, highlighting the compensatory roles for MCL-1 and BCL-XL in cell survival [37,53].

The MCL-1 upregulation is often maintained by oncogenic signaling that stabilizes MCL-1 and preserves mitochondrial stability, despite BCL-2 blockade. In AML samples, the activation of RAS/MAPK signaling has been shown to drive venetoclax resistance via MCL-1, establishing a direct mechanistic link between pathway activation and escape via BCL-2 inhibition [37]. MCL-1 has an intrinsically short half-life, but contemporary literature supports the idea that ERK-mediated phosphorylation, such as at Thr163, can stabilize the protein and extend its half-life, preserving anti-apoptotic buffering under therapy [50,54] Alongside ERK signaling, PI3K/AKT signaling can also stabilize MCL-1 by inhibiting GSK3β-dependent degradation pathways. This AKT–GSK3β axis has been shown to modulate MCL-1 levels and apoptotic sensitivity in leukemic cell models [55]. Finally, in FLT3-dependent AML, downstream STAT5 signaling regulates anti-apoptotic pathways, including the BCL-2 protein family, explaining why kinase pathway inhibition can help restore venetoclax sensitivity [56].

The bone marrow can further promote adaptive resistance by providing cytokines and survival signals that upregulate MCL-1 and activate the pathways discussed above. Signals from the bone marrow, both stromal cell interactions and cytokine pathways such as IL-6 and JAK/STAT, can increase anti-apoptotic protein expression and reduce sensitivity to BCL-2 inhibitors. Thus, venetoclax sensitivity can be driven by the bone marrow microenvironment and not cytogenetics alone [57,58]. Functionally, this implies that cytokines such as GM-CSF and G-CSF can trigger bone marrow-like signaling that helps cancer cells avoid death. Blockade of the JAK pathway has been previously explored in studies as a way to counteract these cytokine-mediated resistance programs that protect leukemic cells from apoptosis [59]. Overall, these results show support for the rationale behind venetoclax combination therapy that lowers MCL-1 or BCL-XL activity or blocks bone marrow signaling, particularly in patients at increased susceptibility to switching of apoptotic dependence [37,52].

### 5.3. Execution Failure

Even if venetoclax is successful in disrupting anti-apoptotic proteins, complete cell death still depends on a functional mitochondrial death machinery. The loss or impairment of key effectors BAX and BAK prevents MOMP, enabling leukemic cells to survive despite BCL-2 inhibition. One CRISPR screening study in AML models showed that the loss of TP53, BAX, and PMAIP1 (NOXA) genes confers venetoclax resistance, linking impaired p53–BH3 signaling and impaired BAX-mediated execution to diminished venetoclax activity [60]. Clinically, patients who relapse after venetoclax therapy have been found to have acquired BAX mutations that cause resistance to BH3 mimetics, showing that failure to execute apoptosis can also be a relapse mechanism [8].

## 6. Therapeutic Strategies Based on Dependence

### 6.1. Venetoclax with Hypomethylating Agents

The standard low-intensity combination regimen pairs venetoclax with a hypomethylating agent, most commonly azacitidine or decitabine. Clinically, results from VIALE-A have shown that this combination regimen improves remission rates and survival time compared to azacitidine alone in older or unfit patients, with this also seen in earlier phase 1B studies [4]. Hypomethylating agents appear to promote apoptotic priming in a way that heightens cell sensitivity to BCL-2 inhibition (Table 1). In patients, this combination targets oxidative phosphorylation and suppresses leukemic stem cells, supporting a synergistic mechanism that goes beyond reducing cell count [5]. Preclinical studies have already demonstrated that azacitidine can induce NOXA (PMAIP1) through a non-epigenetic mechanism, counteracting MCL-mediated resistance and enhancing venetoclax activity [23].

However, resistance develops as leukemic cells can adapt to therapeutic pressure by shifting away from BCL-2 dependence. One common resistance pathway involves increased MCL-1 expression or stabilization, allowing it to sequester pro-apoptotic factors released after BCL-2 inhibition and prevent BAX/BAK activation. Evidence from acquired resistance models and patient-linked analysis shows that RAS/MAPK signaling drives this resistance through MCL-1 upregulation and mitochondrial fitness, directly supporting combination strategies that inhibit this pathway [39]. A second route of resistance is lineage-associated buffering, in which differentiation state can drive a shift away from BCL-2 toward MCL-1 or BCL-XL dependence, leading to venetoclax dependence. Together, these findings indicate that venetoclax with hypomethylating agents is effective when leukemic cells remain dependent on BCL-2 and apoptotic priming pathways are undisturbed, but responses are often short-lived when resistance emerges through MCL-1 or BCL-XL escape, providing a rationale for other combination strategies discussed later [7,37].

### 6.2. Venetoclax with FLT3 Inhibitors

Combining venetoclax with an FLT3 inhibitor is appealing because FLT3 inhibition weakens MCL-1-mediated mitochondrial survival and shifts leukemic cells towards BCL-2 dependence. In a study using FLT3-ITD AML models, inhibition of FLT3 suppressed MCL-1-dependent survival, sensitizing cells to venetoclax-induced apoptosis [56]. Likewise, preclinical studies with the FLT3 inhibitor gilteritinib reveal synergy with venetoclax through suppression of MCL-1, indicating that inhibition of FLT3/AXL signaling disrupts an MCL-1-mediated escape pathway [63]. Clinically, this biology has been translated into meaningful clinical activity, with venetoclax combined with gilteritinib achieving high clinical and molecular response rates in relapsed/refractory cases of FLT3-mutated AML, consistent with restored sensitivity to BCL-2 inhibition [64].

### 6.3. Venetoclax with MCL-1 Inhibitors

The rationale of combining venetoclax with an MCL-1 inhibitor is that MCL-1 often serves as a common escape mechanism, preserving mitochondrial integrity when BCL-2 has been inhibited. By directly inhibiting MCL-1, a key escape mechanism for venetoclax resistance is disrupted, preventing the re-sequestration of pro-apoptotic factors, thereby allowing BAX/BAK activation and MOMP. In AML models, selective MCL-1 inhibitors, such as VU661013 and AMG 176, triggered apoptosis in MCL-1-dependent cells and acted synergistically with venetoclax, including in venetoclax-resistant models and patient-derived xenografts, demonstrating that dual BH3-mimetic therapy can overcome a cell’s compensatory anti-apoptosis pathway [31,35].

Safety remains a key limitation, as MCL-1 plays a critical role in normal tissues, making bone marrow suppression and cardiotoxicity likely on-target risks. Early clinical evaluation of the direct MCL-1 inhibitor AZD5991 revealed a high frequency of troponin elevation and severe cytopenic events, including febrile neutropenia and anemia, underscoring both cardiac monitoring concerns and on-target hematologic toxicity [51]. Cardiac toxicity has also been reported in other MCL-1 inhibitor development programs, including an FDA clinical hold for AMG 397, reinforcing the need for optimized dosing schedules, strict eligibility criteria, and frequent cardiac monitoring in future venetoclax-based combination regimens [51].

### 6.4. BCL-XL Targeting

A subset of AML cases is intrinsically less sensitive to venetoclax due to BCL-XL survival dependence instead of BCL-2. This is most evident in AML cells with erythroid/megakaryocytic differentiation, a relationship between differentiation state and BCL-XL-dependent survival that was discussed earlier [7]. In these lineage-skewed states, BCL-2 inhibition alone is often ineffective, as it does not target the dominant anti-apoptotic protein and instead requires overcoming BCL-XL buffering [7,65]. These findings provide a strong biological rationale for targeting BCL-XL in erythroid and megakaryocytic AML, as well as in other contexts where BCL-XL is dominant [7].

The main challenge is that by directly targeting BCL-XL in leukemic cells, it also causes on-target platelet toxicity due to the essential role that BCL-XL has in platelet survival. Clinical studies with the dual BCL-2/BCL-XL inhibitor navitoclax showed dose-limiting thrombocytopenia, which mechanistic studies have confirmed as an on-target consequence of platelet dependence on BCL-XL [66,67]. Newer therapies have therefore focused on targeting tumor-selective BCL-XL and sparing platelets. One promising strategy is the use of platelet-sparing BCL-XL-targeting PROTACs such as DT2216, which leverage low VHL expression in platelets to mitigate thrombocytopenia and have advanced into the first-in-human studies [68]. Another approach involves using delivery modalities such as antibody-drug conjugates to enrich BCL-XL-directed payloads in tumor cells while reducing systemic platelet exposure [69]. Together, these approaches reflect the emerging strategy of matching BCL-XL-dependent AML phenotypes with BCL-XL-directed therapy while circumventing on-target platelet toxicity and targeting the tumor microenvironment.

### 6.5. Treatment Sequencing and Timing

In clinical practice, the primary constraint for venetoclax-based combinations is treatment-limiting cytopenia, underscoring the importance of timing and duration when designing an appropriate regimen. In VIALE-A, management of cytopenias included protocol-guided treatment interruptions and reduction in venetoclax from 28 to 21 days per cycle, with long-term follow-up confirming that many patients with sustained remission are maintained on 21 days or fewer per cycle [4,70]. This supports the practice of intermittent dosing when combining venetoclax with agents that target MCL-1 or BCL-XL escape, enabling sufficient on-target leukemic cell eradication while allowing hematopoietic recovery and adjusting treatment intensity based on apoptotic dependence (Table 1). Functional monitoring tools can support this approach, as BH3 profiling directly measures mitochondrial priming and anti-apoptotic dependence, thus complementing protein-expression ratios in identifying emerging dependence switching in leukemic cells [42,71]. Lastly, adverse effects specific to each target should guide the selection and scheduling of venetoclax-based combinations. BCL-XL inhibition is limited by platelet-dependent thrombocytopenia, whereas early clinical experience with direct MCL-1 inhibitors highlights the need to monitor cardiac biomarkers such as troponin and overlapping myelosuppression [51,67,68].

### 6.6. Ongoing Clinical Trials Targeting Venetoclax Resistance

As summarized in Table 2, several clinical trials are underway that involve combinations with venetoclax in an effort to overcome or prevent resistance through blocking parallel survival pathways. These include blocking the FLT3–MCL–1 pathway using FLT3 inhibition, transcription programs associated with disease maintenance using menin inhibition, targeting the IDH1 to overcome another abnormality, and inhibiting MCL-1 or BCL-XL to neutralize the alternative survival signaling. All these strategies involve an emerging paradigm of a combination of venetoclax and pathway inhibitors to overcome venetoclax resistance but have not yet been tested clinically [72].

## 7. Biomarkers and Future Therapeutic Directions

### 7.1. Biomarkers to Guide Dependence-Based Therapy

**Figure 3:**
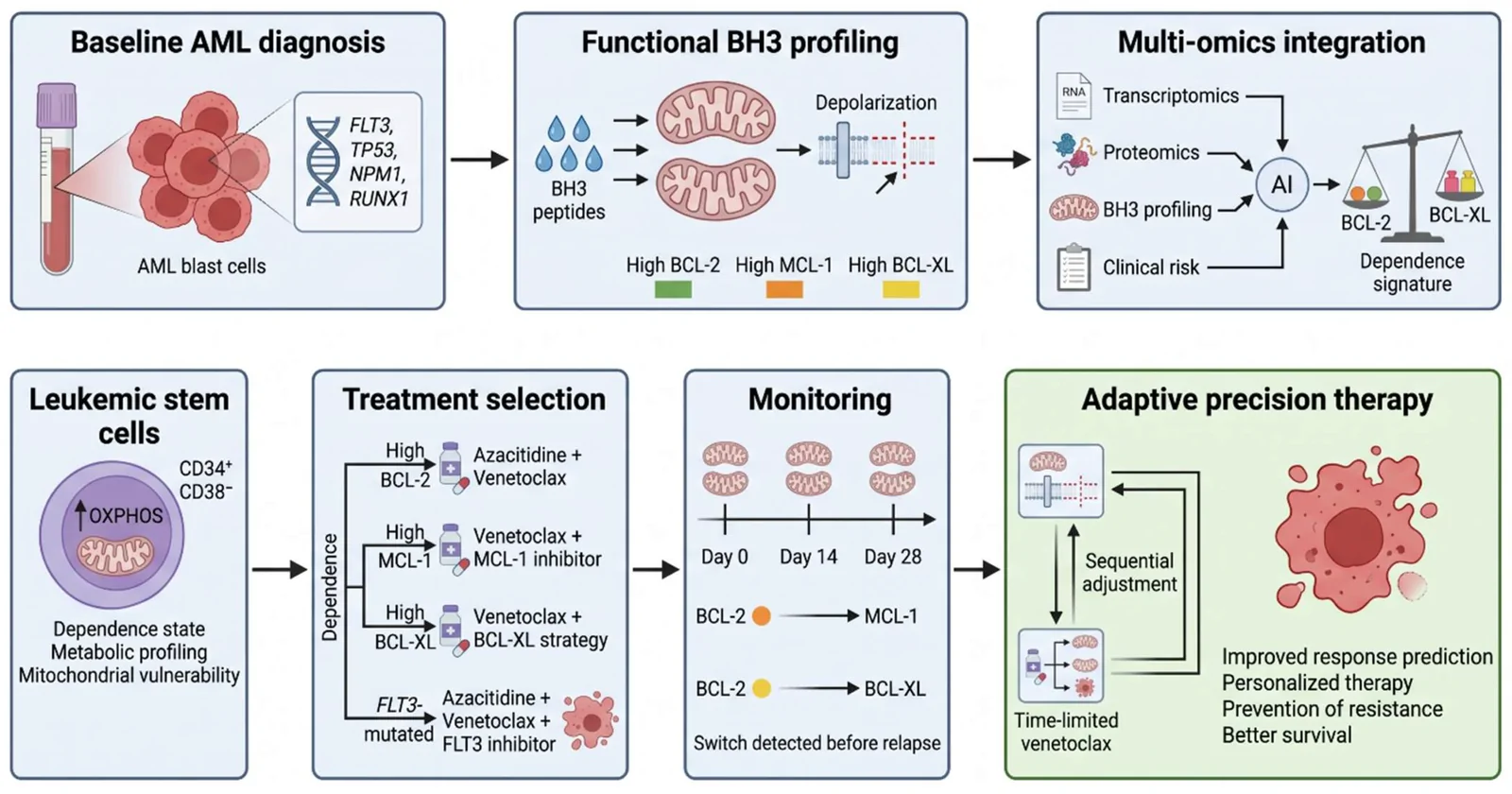
Proposed biomarker-guided adaptive therapeutic framework for AML. Proposed framework integrating genomic biomarkers, functional BH3 profiling, leukemic stem-cell vulnerabilities, multi-omics analysis, and clinical risk to characterize anti-apoptotic dependencies and inform therapeutic selection. Longitudinal monitoring of shifts among BCL-2, MCL-1, and BCL-XL dependence may provide a strategy for adapting therapy as resistance evolves; however, the clinical utility of this approach requires prospective validation [61].

The use of BH3 profiling, also known as functional priming, offers a direct functional measurement of mitochondrial priming and anti-apoptotic dependence that complements cytogenetic data when predicting response to BH3 mimetics. In AML, reduced mitochondrial priming correlates with venetoclax resistance, supporting the use of functional priming as a treatment-relevant biomarker instead of a description of phenotype [47]. More recently, dynamic BH3 profiling has been adapted as a rapid (48 h) ex vivo assay for patient samples. It predicts clinical outcomes by measuring how drugs alter apoptotic priming, helping detect early changes in cellular dependence during therapy [73]. Early clinical studies in frontline azacitidine–venetoclax treatment show that BH3 profiling during therapy can identify treatment responders versus nonresponders, emphasizing its applicability in clinical settings. Together, these approaches provide a framework for integrating functional apoptotic profiling with molecular and clinical features to guide biomarker-informed treatment selection and longitudinal adaptation (Figure 3).

Given that dependence is commonly distributed across multiple BCL-2 family proteins, clinically useful biomarkers are increasingly focused on multi-protein patterns rather than single markers. One notable example is a study from Waclawiczek, A. et al., that demonstrated that combining transcriptional, proteomic, functional, and clinical data can predict response to azacitidine–venetoclax treatment through coordinated BCL-2 family expression in AML stem cells [9]. Using this approach, clinically practical readouts rely on relative measures of BCL-2 versus MCL-1 or BCL-XL to identify the dominant anti-apoptotic protein, providing an alternative to full functional priming while still capturing underlying dependence [9].

As the risk of relapse is often driven by leukemic stem cells and the mitochondrial pathways, they rely on biomarkers that target stem and progenitor cells to better identify treatment vulnerabilities than profiling leukemic cells alone. Studies have shown that venetoclax combined with azacitidine can disrupt energy metabolism and oxidative phosphorylation and selectively target leukemic stem cells. These effects provide a mechanistic basis for using leukemic stem cell dependency and metabolic profiling as tools to predict treatment response [5,74]. In line with these findings, when dependency signatures such as the BCL-2 family proteins were measured in AML cells, they were found to correlate with clinical response to azacitidine and venetoclax [9]. This supports the idea of a future approach in which baseline and treatment leukemic stem cell dependence states are measured to guide both initial therapy selection and adaptation as resistance emerges [9].

### 7.2. Future Directions

An important next step is the development of prospective trials that move beyond single-factor risk grouping to jointly integrate cytogenetic risk with functional anti-apoptotic dependence. Existing frameworks already classify genetic risk across treatment intensities, including hypomethylating agent and venetoclax regimens, creating a consistent structure for cytogenetic grouping. Advances now make it possible to incorporate functional measures of anti-apoptotic dependence, including stem and progenitor BCL-2 family signatures or priming assays, to distinguish treatment states within each genetic risk category. A stem cell-focused combinatorial BCL-2 family expression biomarker that is predictive of azacitidine–venetoclax treatment response illustrates how dependence states can be measured and used to guide clinical trial design [9]. Early clinical experience shows that targeted therapies can be added to an azacitidine–venetoclax backbone for specific molecular subgroups, enabling stratified approaches that can be applied without moving away from the established standard treatments [75].

Another promising approach is early identification of dependency switching during therapy, with treatment intensity adjusted according to predefined rules. Dynamic BH3 profiling provides a rapid functional test that measures treatment-induced changes in apoptotic sensitivity and predicts response to chemotherapy and targeted therapies, enabling early detection of shifts toward MCL-1 or BCL-XL dependence before relapse becomes clinically apparent [73]. Clinical trials incorporating these early functional readouts must also address treatment tolerance. Venetoclax-based regimens commonly require adjustments in dose scheduling due to cytopenia risk, with evidence from real-world studies and VIALE-A suggesting that shorter or intermittent venetoclax exposure can preserve efficacy while reducing toxicity [76,77]. This supports a treatment paradigm that integrates dependency-aligned drug combinations with sequenced or time-limited dosing and targeted monitoring to maximize leukemic cell death while limiting toxicity and other on-target effects.

## 8. Conclusions

Cytogenetic abnormalities, together with their phenotypic maturation, offer a useful framework for interpreting the regulatory mechanisms of mitochondrial apoptosis in AML. The presence of certain chromosomal arrangements and other high-risk cytogenetic patterns causes unique transcriptional and signaling pathways that impact mitochondrial priming, altering the balance of translation and apoptosis within the BCL-2 family. Modern classification and risk-stratification guidelines reflect this biology by recognizing many chromosomal abnormalities as both disease-defining and prognostically significant. These same features also help explain the variability in venetoclax response across the different AML subtypes.

Resistance to BCL-2 inhibition most commonly arises through a shift in anti-apoptotic dependence, rather than a single uniform mechanism. When leukemic cells are treated with venetoclax, they can shift their survival dependence to alternative anti-apoptotic proteins, particularly MCL-1 or BCL-XL. These adaptations allow them to re-sequester pro-apoptotic activators and block BAX/BAK-mediated mitochondrial outer membrane permeabilization. Previous studies have clarified that RAS/MAPK pathway activation promotes MCL-1-mediated resistance. Concurrently, phenotypic studies have shown that monocytic differentiation is associated with reduced BCL-2 dependence, while erythroid and megakaryocytic differentiation is more reliant on BCL-XL. Overall, both patterns correlate with decreased efficacy of venetoclax.

Further research is needed into possible clinical applications of changes in dependency on anti-apoptotic proteins from BCL-2 to other factors. The drug venetoclax can become a basis for the combination therapy directed at major forms of resistance to treatment. However, the dose, schedule of administration, and drugs in combination should be clarified by further clinical trials. Although the combination of azacitidine and venetoclax shows promising results, the cytopenia caused by the therapy presents a major drawback, which requires a personal approach to choosing the dose and schedule. New technologies including BH3 profiling, multiple dependence signatures, and stem- and progenitor-cell profile show potential in the identification of apoptotic dependencies and possible changes in such dependencies during the therapy. However, the clinical utility of these biomarkers and their ability to guide treatment selection or prevent relapses have not yet been fully established and require prospective clinical validation (Figure 3). Such studies will be essential to determine whether dependence-based, biomarker-informed strategies can improve the durability of responses and clinical outcomes in AML.

## Figures and Tables

**Table 1:** Therapeutic strategies targeting anti-apoptotic dependence and venetoclax resistance in acute myeloid leukemia [4,32,61,62].

Drug/Class	Target	Mechanism Relevant to Venetoclax	AML Subgroup	Key Findings
Venetoclax	BCL-2	Releases BAX/BAK-mediated apoptosis	Broad AML	Backbone low-intensity therapy
Azacitidine	DNA methylation/OXPHOS	Primes apoptosis, induces NOXA	Older/unfit AML	Synergistic with venetoclax
Gilteritinib	FLT3/AXL	Suppresses MCL-1 signaling	FLT3-mut AML	Restores venetoclax sensitivity
Quizartinib	FLT3	Reduces FLT3-driven survival	FLT3-ITD AML	Rational combo partner
AMG-176	MCL-1	Blocks compensatory MCL-1 escape	Venetoclax-resistant AML	Strong preclinical synergy
VU661013	MCL-1	Dual BH3 targeting	Resistant AML	Overcomes adaptive resistance
Revumenib	Menin–KMT2A	Disrupts HOXA/MEIS1 programs	KMT2A-r AML	Rational triplet strategy
DT2216	BCL-XL PROTAC	Targets BCL-XL with platelet sparing	Erythroid/megakaryocytic AML	Reduced thrombocytopenia
Navitoclax	BCL-2/BCL-XL	Dual inhibition	BCL-XL-dependent AML	Limited by thrombocytopenia
JAK inhibitors	JAK/STAT	Blocks cytokine-mediated resistance	Microenvironment-driven resistance	Sensitizes to venetoclax
MAPK inhibitors	RAS/MAPK	Prevents MCL-1 stabilization	RAS-driven AML	Reduces adaptive resistance

**Table 2:** Several ongoing clinical trials evaluate venetoclax-based combinations designed to prevent or overcome resistance by targeting complementary survival pathways.

Clinical Trial (NCT Number)		Phase	Population/AML Subset	Therapeutic Strategy (Venetoclax Combination)	Mechanism/Rationale to Overcome Venetoclax-Mediated Resistance	Venetoclax Resistance Mechanisms Addressed	Current Status (As of Aug 2026)
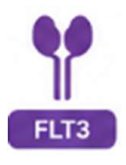	**NCT05010122**	**I/II**	**Newly diagnosed, relapsed/refractory, or refractory** ***FLT3-mutated* AML/MDS**	**Venetoclax + gilteritinib** **+** **Decitabine/Cedazuridine** **(ASTX727)**	**FLT3 inhibition suppresses FLT3-driven survival signaling, reduces MCL-1 upregulation and leukemic stem cell survival, and may restore/enhance BCL-2 dependence.**	**• Upregulation of MCL-1** **• FLT3-dependent survival signaling** **• Leukemic stem cell persistence**	**Recruiting** **(Active, not** **recruiting complete)**
** 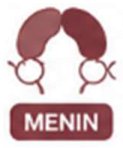 **	**NCT06284486**	**I/II**	**AML with measurable residual disease** ***(NPM1-* mutated *, KMT2A-*** **rearranged, or *NUP98-* rearranged)**	**Venetoclax + revumenib** **(menin inhibitor)**	**Dual inhibition of BCL-2 and menin-dependent transcriptional programs targets residual disease and may prevent/overcome relapse after venetoclax therapy.**	**• Persistence of residual disease** **• Transcriptional reprogramming (menin-driven)** **• Microenvironment-mediated survival**	**Recruiting** **(Active, not** **recruiting complete)**
** 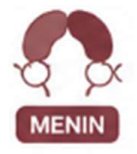 **	**NCT06652438** **(HO177)**	**III**	**Newly diagnosed *NPM1-* mutated or *KMT2A-* rearranged AML, ineligible for intensive chemotherapy**	**Azacitidine + venetoclax** **+ revumenib**	**Adding menin inhibition to the established VEN-AZA backbone targets molecularly defined leukemic survival programs to improve response depth and durability.**	**Molecular relapse after VEN-AZA** **• Menin-driven survival pathways** **• Clonal persistence**	**Recruiting** **(Active, not** **recruiting complete)**
** 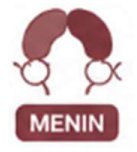 **	**NCT06852222** **(cAMeLot-2)**	**III**	**Newly diagnosed *NPM1*-mutated or *KMT2A-* rearranged AML, ineligible for intensive chemotherapy**	**Azacitidine + venetoclax** **+ bleximenib** **(menin inhibitor)**	**Menin inhibition combined with BCL-2 blockade to prevent persistence and relapse associated with venetoclax resistance.**	**• Menin-driven transcriptional programs** **• Minimal residual disease** **• Acquired resistance/relapse**	**Recruiting** **(Active, not** **recruiting complete)**
** 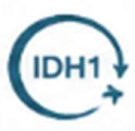 **	**NCT07032727**	**II**	**Relapsed/refractory *IDH1-*mutated myeloid malignancies with co-signaling mutations**	**Olutasidenib +** **cladribine/LDAC ± venetoclax** **or** **olutasidenib + gilteritinib** **± venetoclax**	**Targeting IDH1 mutation with or without venetoclax and/or pathway-directed therapy to counteract cooperating signaling abnormalities that drive venetoclax resistance.**	**• Co-occurring signaling mutations** **• Pathway activation (e.g., FLT3, RAS)** **• Metabolic and survival reprogramming**	**Recruiting** **(Active, not** **recruiting complete)**

Abbreviations: AML, acute myeloid leukemia; MDS, myelodysplastic syndrome; VEN, venetoclax; LDAC, low-dose cytarabine; FLT3, FMS-like tyrosine kinase 3; MCL-1, myeloid cell leukemia-1; BCL-2, B-cell lymphoma 2; NPM1, nucleophosmin 1; KMT2A, lysine methyltransferase 2A; NUP98, nucleoporin 98. Note: Trials listed are ongoing as of August 2026 (ClinicalTrials.gov). Trial status may change over time.

## Data Availability

No new data were created or analyzed in this study. Data sharing is not applicable to this article.
